# Retinol intake is associated with the risk of chronic kidney disease in individuals with type 2 diabetes mellitus: results from NHANES

**DOI:** 10.1038/s41598-023-38582-z

**Published:** 2023-07-18

**Authors:** Rong Ma, Chunpeng Xie, Shaoqing Wang, Xiang Xiao

**Affiliations:** 1 People’s Hospital of Xindu District, Chengdu, 610500 China; 2 Department of Nephrology, The First Affiliated Hospital of Chengdu Medical College, No. 278, Middle Section of Baoguang Avenue, Xindu District, Chengdu, 610500 Sichuan China; 3 The Second Affiliated Hospital of Chengdu Medical College, Chengdu, 610000 China

**Keywords:** Diseases, Endocrinology, Nephrology

## Abstract

The aim of this study was to investigate the potential association between retinol intake and the risk of chronic kidney disease (CKD) in individuals with type 2 diabetes mellitus (T2DM). The study included individuals diagnosed with T2DM between 2009 and 2018 from the NHANES database. Demographic and laboratory test data were collected for these individuals, as well as information on CKD diagnosis. Logistic regression models were utilized to estimate the relationship between different retinol intakes and the risk of CKD in patients with T2DM. A total of 3988 patients were included in the study. The mean prevalence of CKD in the T2DM population in the United States from 2009 to 2018 was 36.98 (0.02)%. Multivariate logistic regression analysis revealed a 26% decrease in the incidence of CKD in individuals with higher retinol intake compared to those with lower retinol intake in T2DM (OR = 0.74; 95% CI 0.56–0.98). Furthermore, an increase in retinol intake per 1-standard deviation (SD) was associated with a 16% decreased risk of the incidence of CKD (OR = 0.84; 95% CI 0.72–0.97). Lower retinol intake is an independent risk factor for the onset of CKD in patients with T2DM, and augmenting moderate quantities of retinol confers potential nephroprotective advantages.

## Introduction

National surveillance data from the United States shows that between 2015 and 2018, 15% of the adult population in the country suffered from chronic kidney disease (CKD). The burden of CKD is particularly high in diabetes mellitus (DM) patients, with approximately 40% of type 2 diabetes mellitus (T2DM) patients and 30% of type 1 diabetes mellitus (T1DM) patients suffering from CKD^1,2^. Given the significant increase in DM prevalence and the accompanying high rate of kidney failure, the persistent high incidence of CKD in the United States is concerning^3,4^. The trend of new CKD among U.S. patients with DM has been increasing annually from 2015 to 2020^5^.

Medical Nutritional Therapy (MNT) is the cornerstone of the multi-disciplinary management of DM, it helps to manage DM and prevent complications^6,7^. Emerging evidence suggests that dietary considerations play a crucial role in individuals with DM suffered from CKD^8^. Retinol, the active form of vitamin A, is essential for various biological functions, including proliferation, apoptosis, differentiation, and metabolism^9^. The primary dietary sources of retinol are beta-carotene from plants and retinyl esters from animal products.In the intestinal epithelium, retinol can be converted to retinyl esters and packaged into chylomicrons. In the liver, retinyl esters are cleared from chylomicrons and can be stored or metabolized to retinol and retinol derivatives^10^. Mammalian cells absorb retinol through STRA6, and the retinol can be metabolized to retinal and eventually to retinoic acid (RA)^11^.

Numerous studies have shown a significant correlation between retinol and its metabolite RA levels in the body and the development of DM and its complications. Female rats (8–10 weeks old) fed a high-fat diet for 6 weeks developed obesity, hyperglycaemia and insulin resistance, and interestingly the administration of Vitamin A (VA) for 2 weeks completely corrected the hyperglycaemia and hyperinsulinemia in the fasted state^12^. The beneficial effect of treatment with retinoic acid (RA), a metabolite of retinol, which reduces blood glucose levels in Zucker diabetic fatty (ZDF) rats from 528 to 223 mg/dL, was attributed to the inhibition of nuclear factor kappa-B (NF-kB) signaling and its associated inflammatory response^13^. RA-treated female ob/ob mice lost weight and had improved results in intraperitoneal glucose and insulin tolerance tests^14^. Studies have demonstrated a lower proportion of insulin resistance in subjects in the upper quartile of retinol compared to the lower quartile^15,16^. A negative association was observed between higher dietary retinol or retinol equivalent intake and the risk of diabetic retinopathy, with each 100 μg/d increase in dietary retinol equivalent associated with a 12% reduction in risk^17^. Retinol metabolism disorder was involved in the development of myocardial injury in T2DM and the injury was improved with retinol intake^18^.

More and more evidence had indicated the involvement of retinol and its metabolite RA in the incidence and progress of diabetic kidney disease (DKD). In the kidneys of streptozotocin (STZ)-induced diabetic rats, RA treatment decreased the expression of inflammatory factors and the incidence of proteinuria^19^. Similarly, a study in STZ-induced diabetic mice with diabetic nephropathy at 16 weeks of age (4 weeks after STZ injection), intraperitoneal administration of RA (15 mg/kg body weight, 3 times a week) for 8 weeks decreased urinary albumin and protein excretion^20^. In a high-fat diet-induced diabetes nephropathy mouse model, treatment with a RARβ2-specific agonist for 12 weeks reduced proteinuria and urinary albumin excretion and induced expression of podocin and Wilms tumour-suppressor gene 1 in podocytes^21^. However, to date, no studies have explored the relationship between retinol and the risk of CKD in individuals with T2DM.

Therefore, this study aims to investigate the association between retinol intake and the risk of CKD in individuals with T2DM using the NHANSE database.

## Materials and methods

### Study design and individuals

The National Health and Nutrition Examination Survey (NHANES) is an ongoing study that provides valuable health-related data on adults and children in the United States. The survey enrolls a representative sample of the U.S. population using a stratified, multi-stage probability design and conducts every 2 years. The NHANES interview includes questions on demographics, socioeconomics, diet, and health-related issues. The examination component includes medical, dental, and physiological measurements, as well as laboratory testing conducted by trained medical personnel. Data were obtained through structured interviews with individuals in their homes, health screenings at mobile examination centers, and sample analysis in the laboratory, and the diet data is based on a two-day recall survey^22^. The objective of the dietary interview component is to obtain detailed dietary intake information from NHANES participants. The dietary intake data are used to estimate the types and amounts of foods and beverages (including all types of water) consumed during the 24-h period prior to the interview (midnight to midnight), and to estimate intakes of energy, nutrients, and other food components from those foods and beverages, we extracted the total two-day retinol intake for the participating individuals from 2009 to 2018.

**Figure 1:**
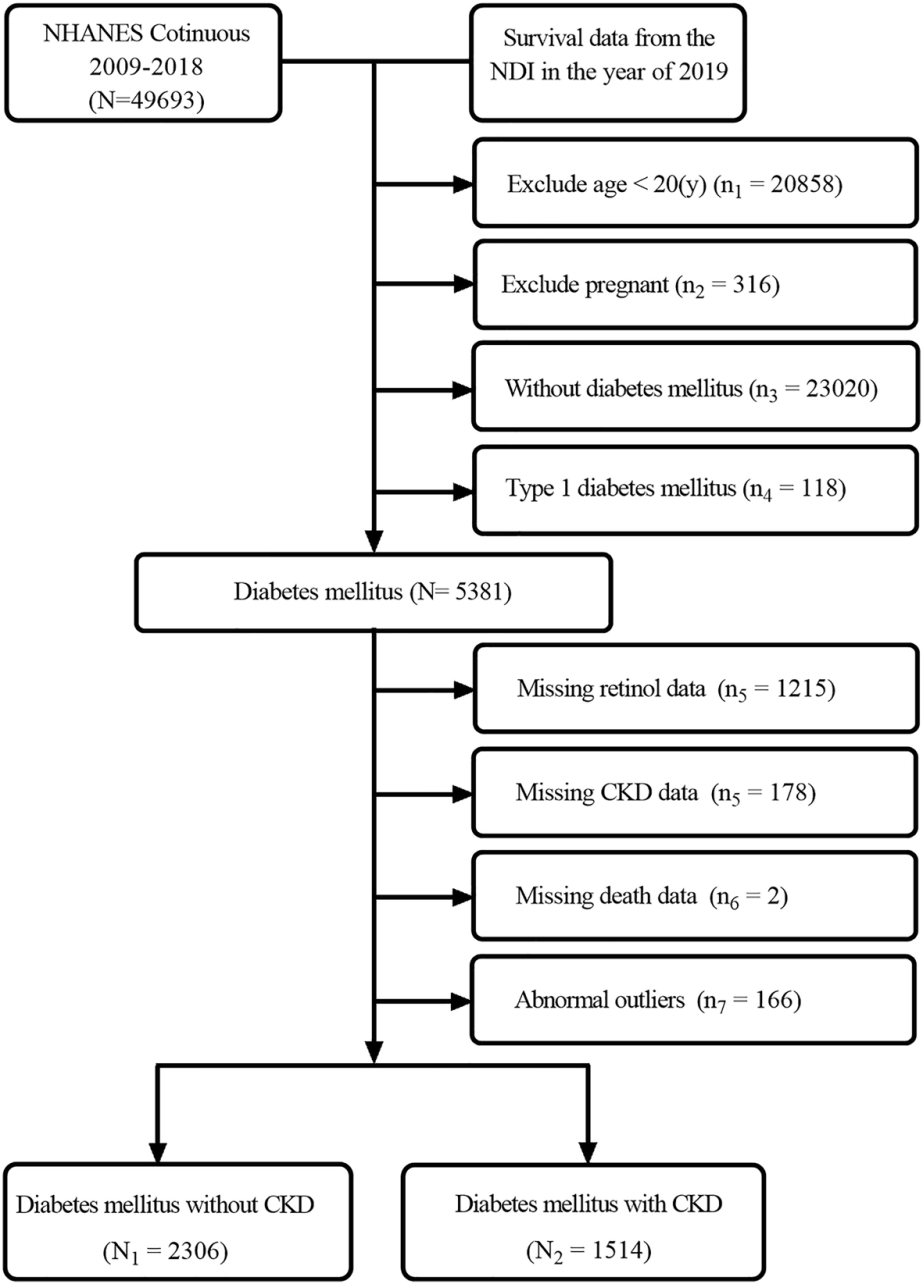
Flowchart of included individuals in this study.

To ensure accuracy, we used specific diagnostic criteria for diabetes and chronic kidney disease (CKD). The diagnostic criteria for diabetes were 1) doctor told you to have diabetes, 2) HbA1c (%) > 6.5, 3) fasting glucose (mmol/l) ≥ 7.0, 4) random blood glucose (mmol/l) ≥ 11.1, and 5) two-hour oral glucose tolerance test (OGTT) blood glucose (mmol/l) >  = 11.1, meet any of the above conditions^17^. Those with albumin-to-creatinine ratio (ACR) above 30 mg/g or estimated glomerular filtration rate (e-GFR) below 60 mL/min/1.73 m^2^ were defined as patients with CKD^23^. Exclusion criteria were: (1) age < 20 years, (2) pregnant, (3) type 1 diabetes (defined as a diagnosis of diabetes at age < 30 years and the insulin use as the only hypoglycemic agent), (4) without diagnostic information on CKD and diabetes, and (5) missing complete two-day diet recall data (Fig. 1). Definitions or criteria for the diagnosis of smoker, alcohol user, hypertension, anemia, hyperlipidemia, etc. are detailed in Supplementary Table 1. The baseline was defined as the time point of population enrollment in the NHANSE study. In addition, rigorous laboratory analyses were performed, including ACR, e-GFR, HbA1c, and serum albumin at baseline. The further details of these measurements are documented in the NHANES Laboratory Medical Technician Procedure Manual^24^. The study protocol conformed to the ethical standards of the 1964 declaration of Helsinki and its later amendments. All procedures involving human participants were approved by the National Center for Health Statistics Research Ethics Review Committee, and all participants signed informed consent forms.

### Statistical analysis

In accordance with CDC guidelines, all analyses considered weighted samples and designed stratification and clustering to derive estimates for the general US population. Weighted means (standard errors (SE)) were used to express continuous variables, while percentages were used for categorical variables. Between-group differences were assessed using weighted t-tests for continuous variables and weighted chi-square tests for categorical variables. The effect of changes in retinol intake on the risk of CKD in individuals with T2DM was determined using restricted cubic spline plots, and individuals with different retinol intakes were divided into two groups using the dichotomous method. The relationship between retinol intake and clinical parameters was analyzed using weighted linear regression. Additionally, the relationship between retinol intake and the risk of CKD in individuals with T2DM was analyzed using weighted logistic regression, and the consistency of the results was assessed by subgroup analysis. We calculate the weights based on the included variables, finding the subset of variables with the smallest number, selecting their corresponding weights, and finally combining the weights for multiple years. For our study, we chose weights with a two-day dietary log, then weights from multiple years 2009–2018 were merged. The missing values are being processed using the deletion method. All statistical tests were performed using R 4.2.2. A two-sided *P <* 0.05 was considered statistically significant.

## Results

### Baseline characteristics

The data of 49,693 individuals registered in the NHANES database from 2009 to 2018 were included. After final screening, a total of 3,820 individuals with diabetes who met the inclusion–exclusion criteria were enrolled, of which 39.7% (1,514/3,820) (unweighted) had coexistent CKD (Fig. 1). The mean prevalence of CKD in the T2DM population in the United States from 2009 to 2018 was 36.98(0.02)%. The mean retinol intake in individuals with T2DM was 726.20(13.53) (mcg). Retinol intake differed significantly between individuals with T2DM without CKD and those with CKD (746.35 (17.26) vs. 691.87 (18.35), *P* = 0.02) (Table 1). Based on the dichotomous method used the cut-off point for retinol intake is set at 609 mcg. Retinol intake was higher in older individuals (60.63(0.46) vs. 59.10(0.48), *P* = 0.005). There were also sex and race differences in retinol intake, with males having a relatively higher intake and race variation potentially related to differences in dietary habits (*P <* 0.001). Individuals with alcohol use and those without anemia had a higher retinol intake (*P <* 0.05) (Table 2).

**Table 1:** Baseline clinical features of enrolled T2DM patients with and without CKD.

Variable	Total (N = 3820)	T2DM without CKD (N_1_ = 2306)	T2DM with CKD (N_2_ = 1514)	P-value
Retinol (μg)	726.20 (13.53)	746.35 (17.26)	691.87 (18.35)	0.02
Age (years)	59.92 (0.39)	56.89 (0.49)	65.07 (0.51)	< 0.001
Serum albumin (g/L)	41.39 (0.11)	41.83 (0.11)	40.63 (0.17)	< 0.001
HbA1c (%)	7.09 (0.04)	6.92 (0.05)	7.38 (0.07)	< 0.001
ACR mg/g)	114.42 (11.35)	10.14 (0.19)	296.36 (29.47)	< 0.001
e-GFR (ml/min/1.73m^2^)	83.14 (0.65)	92.01 (0.58)	67.67 (1.18)	< 0.001
Sex (%)				0.53
Female	50.93 (0.02)	51.69 (1.84)	49.63 (2.13)	
Male	49.07 (0.02)	48.31 (1.84)	50.37 (2.13)	
BMI (%)				0.38
Underweight	0.25 (0.00)	0.17 (0.07)	0.42 (0.15)	
Normal weight	10.35 (0.01)	10.34 (1.05)	10.74 (0.98)	
Overweight	24.10 (0.01)	25.44 (1.37)	22.63 (1.71)	
Obesity	64.00 (0.03)	64.05 (1.59)	66.22 (1.86)	
Race(%)				0.03
Mexican American	10.59 (0.01)	11.03 (1.45)	9.84 (1.31)	
Non-Hispanic	13.93 (0.01)	13.74 (1.36)	14.25 (1.36)	
Black				
Non-Hispanic	59.71 (0.03)	58.23 (2.35)	62.24 (2.33)	
White				
Other Hispanic	6.77 (0.01)	7.84 (0.90)	4.93 (0.70)	
Other Race—Including Multi-Racial	9.01 (0.01)	9.16 (0.76)	8.75 (1.07)	
Anemia (%)				< 0.001
No	86.66 (0.03)	91.41 (0.75)	80.05 (1.70)	
Yes	12.68 (0.01)	8.59 (0.75)	19.95 (1.70)	
Hyperlipidemia (%)				0.37
No	11.51 (0.01)	12.11 (1.12)	10.48 (1.22)	
Yes	88.49 (0.03)	87.89 (1.12)	89.52 (1.22)	
Hypertension (%)				< 0.001
No	27.95 (0.02)	33.65 (1.66)	18.24 (1.51)	
Yes	72.05 (0.03)	66.35 (1.66)	81.76 (1.51)	
Alcohol use (%)				0.004
No	13.00 (0.01)	12.51 (0.90)	17.02 (1.38)	
Yes	78.87 (0.03)	87.49 (0.90)	82.98 (1.38)	
Smoke (%)				0.01
No	50.32 (0.02)	53.01 (1.75)	45.77 (2.16)	
Yes	49.66 (0.02)	46.99 (1.75)	54.23 (2.16)	
Antidiabetic drugs (%)				< 0.001
OHAS	43.51 (0.02)	77.26 (1.65)	63.11 (2.02)	
Insulin	6.61 (0.01)	7.39 (1.18)	15.57 (1.39)	
OHAS + Insulin	10.90 (0.01)	15.35 (1.59)	21.32 (1.88)	

ACR, albumin-creatinine ratio; e-GFR, estimated glomerular filtration rate; BMI, Body Mass Index; CKD, chronic kidney disease; T2DM, type 2 diabetes mellitus.

**Table 2:** Baseline clinical features of enrolled T2DM patients with different retinol intake.

Variable	Total (N = 3820)	Lower retinol intake (0 < intake ≤ 609) (n_1_ = 1916)	Higher retinol intake (609 < intake ≤ 1911) (n_2_ = 1904)	*P*-value
Retinol (mcg)	726.20 (13.53)	367.93 (6.53)	1038.80 (12.75)	< 0.001
Age (years)	59.92 (0.39)	59.10 (0.48)	60.63 (0.46)	0.005
BMI (kg/m^2^)				0.21
Normal-weight	0.25 (0.00)	0.43 (0.12)	0.10 (0.05)	
Obesity	10.35 (0.01)	10.21 (0.91)	10.73 (1.09)	
Over-weight	24.10 (0.01)	23.09 (1.41)	25.58 (1.43)	
Under-weight	64.00 (0.03)	66.26 (1.86)	63.59 (1.72)	
Serum albumin (g/L)	41.39 (0.11)	41.19 (0.15)	41.57 (0.13)	0.05
HbA1c (%)	7.09 (0.04)	7.09 (0.06)	7.08 (0.05)	0.83
Sex				< 0.001
Female	50.93 (0.02)	57.6 (1.83)	45.10 (1.70)	
Male	49.07 (0.02)	42.39 (1.83)	54.90 (1.70)	
Race				< 0.001
Mexican American	10.59 (0.01)	12.22 (1.51)	9.17 (1.36)	
Non-Hispanic	13.93 (0.01)	17.55 (1.72)	10.77 (1.03)	
Black				
Non-Hispanic	59.71 (0.03)	51.33 (2.61)	67.02 (2.14)	
White				
Other Hispanic	6.77 (0.01)	7.50 (1.00)	6.13 (0.74)	
Other Race—Including Multi-Racial	9.01 (0.01)	11.40 (0.97)	6.91 (0.72)	
Hypertension				0.52
No	27.95 (0.02)	27.24 (1.74)	28.58 (1.57)	
Yes	72.05 (0.03)	72.76 (1.74)	71.42 (1.57)	
Anemia				0.03
No	86.66 (0.03)	85.26 (1.22)	88.95 (1.04)	
Yes	12.68 (0.01)	14.74 (1.22)	11.05 (1.04)	
Hyperlipidemia				0.28
No	11.51 (0.01)	12.51 (1.13)	10.63 (1.17)	
Yes	88.49 (0.03)	87.49 (1.13)	89.37 (1.17)	
Alcohol user				0.04
No	13.00 (0.01)	16.13 (1.24)	12.45 (1.14)	
Yes	78.87 (0.03)	83.87 (1.24)	87.55 (1.14)	
Smoking				0.94
No	50.32 (0.02)	50.24 (1.88)	50.41 (1.73)	
Yes	49.66 (0.02)	49.76 (1.88)	49.59(1.73)	
Anti-diabetic drugs				0.30
OHAS	43.51 (0.02)	73.41 (1.92)	69.54 (1.83)	
Insulin	10.90 (0.01)	16.62 (1.81)	18.91 (1.36)	
OHAS + Insulin	6.61 (0.01)	9.98 (1.05)	11.55 (1.17)	
CKD				0.04
No	63.02 (0.02)	60.16 (2.20)	65.52 (1.51)	
Yes	36.98 (0.02)	39.84 (2.20)	34.48 (1.51)	

BMI, Body Mass Index; OHAS, Oral hypoglycaemic agents; T2DM, type 2 diabetes mellitus; CKD, chronic kidney disease.

### Retinol intake associated with clinic features

We evaluated the relationship between retinol intake and clinical parameters. After adjusting for age, sex, race, and BMI using linear regression models, the results showed that retinol had no significant effect on ACR, e-GFR, serum albumin, hemoglobin, or HbA1c (*P* > 0.05) (Table 3).

**Table 3:** Relationship between retinol intake and clinical indicators.

Variables	Adjusted		
	β	95% CI	*P* value
Hemoglobin	0.00	(0.00, 0.00)	0.47
Serum albumin	0.00	(0.00, 0.00)	0.86
ACR	0.01	(− 0.05, 0.06)	0.80
e-GFR	0.00	(0.00, 0.00)	0.06
HbA1c	0.00	(0.00, 0.00)	0.84

Adjust for age (< 65, ≥ 65), sex (‘Female’, ‘Male’), race, BMI. ACR, albumin-creatinine ratio; e-GFR, estimated glomerular filtration rate; BMI, Body Mass Index.

### Risk assessment for the risk of CKD in individuals with T2DM

**Figure 2:**
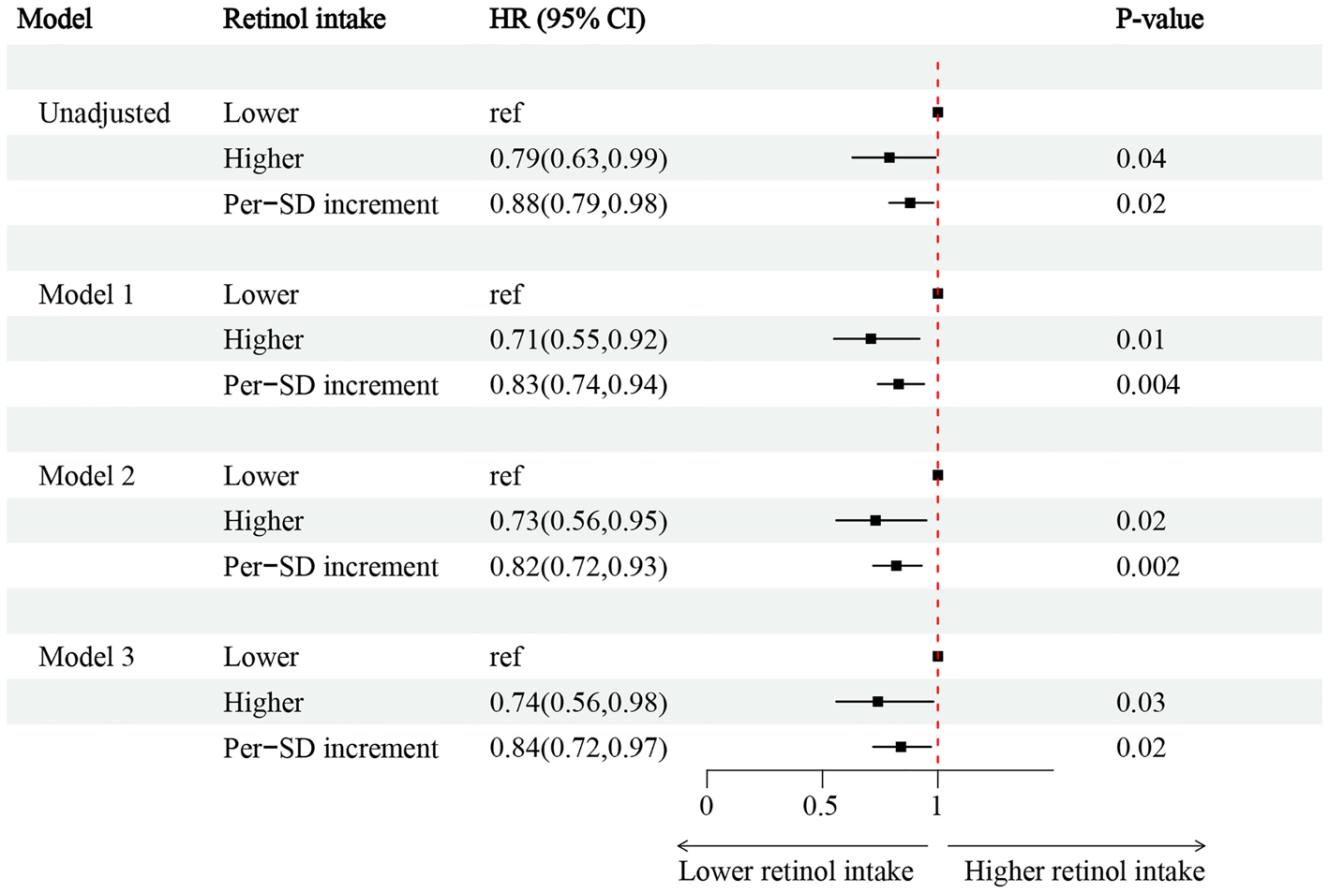
Associations between retinol intake and the occurrence of CKD in individuals with T2DM. **Model 1**^**a**^ adjusted for baseline age, sex, race, BMI; **Model 2**^**b**^ adjusted for covariates in model 1 plus smoking (‘yes’ or ‘no’), alcohol use (‘yes’ or ‘no’), hypertension (‘yes’ or ‘no’). **Model 3**^**c**^ adjusted for covariates in model 2 plus anemia (‘yes’ or ‘no’), anti-diabetic drugs, HbA1c, serum albumin. OR, odds ratio; CI, Confidence interval; BMI, Body Mass Index; CKD, chronic kidney disease; T2DM, type 2 diabetes mellitus.

**Figure 3:**
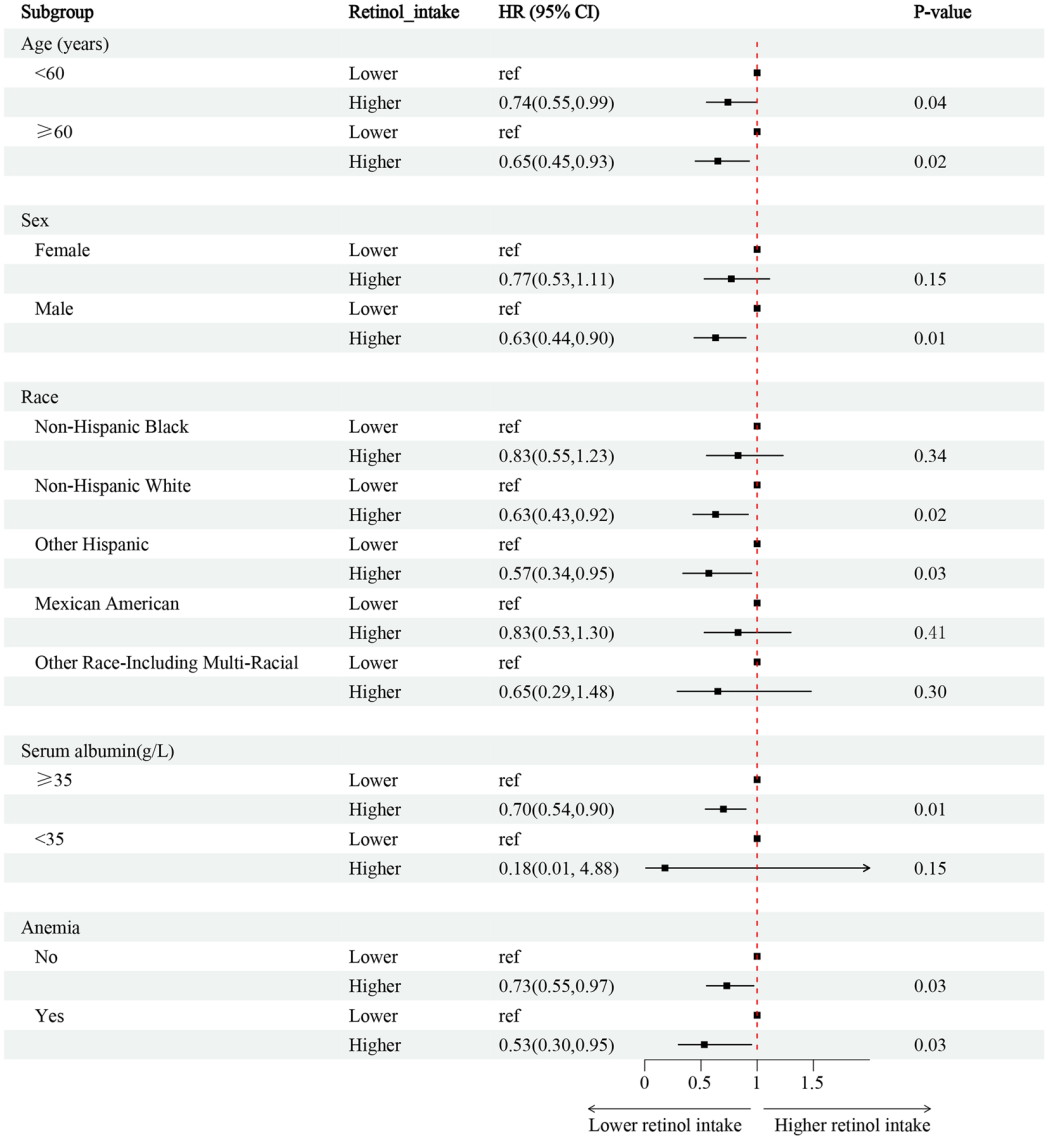
Subgroup analysis of the risk of the occurrence of CKD in individuals with T2DM. Adjust for age (< 65, ≥ 65), sex (‘Female’, ‘Male’), race, BMI, smoke (‘no’, ‘yes’), alcohol use (‘no’, ‘yes’). HR, hazard ratio; CI, confidence interval; BMI, Body Mass Index.

Among 3820 T2DM individuals, 39.7% (1514/3820) of T2DM individuals with CKD, while 60.3% (2306/3820) were without CKD (Fig. 1). Individuals were grouped according to the dichotomous method into lower and higher retinol intake groups. The lower retinol intake group had a retinol intake of (0–609) mcg for 1916 people, and the higher retinol intake group had a retinol intake of (609–1911) mcg for 1904 people. The incidence of CKD was 39.84(2.20)% in T2DM individuals with lower retinol intake, 34.48(1.51) (%) in the higher retinol intake after weighting (Table 2). The association between retinol intake levels and the presence or absence of CKD in T2DM was analyzed using restricted cubic spline plots, which showed that the risk of CKD in individuals with T2DM decreased with increased retinol intake (Supplementary Fig. 1). After adjusting for age, sex, race, Body Mass Index (BMI), smoking (‘yes’ or ‘no’), alcohol use (‘yes’ or ‘no’), hypertension (‘yes’ or ‘no’), hemoglobin, anti-diabetic drugs, HbA1c, serum albumin, energy intake, multivariate logistic regression analysis showed that individuals with higher retinol intake had a 26% decrease in the incidence of CKD compared to those with lower retinol intake in T2DM (OR = 0.74; 95% CI 0.56–0.98). An increase in retinol intake per 1-standard deviation (SD) was associated with a 16% increased risk of CKD incidence (OR = 0.84; 95% CI 0.72–0.97) (Fig. 2, Supplementary-Table 2–3). Subgroup analysis revealed that lower retinol intake was a risk factor for the risk of CKD in individuals with T2DM in the male, serum albumin ≥ 35 g/L, Non-Hispanic White, and Other Hispanic subgroups (Fig. 3).

## Discussion

This study is the first to investigate the association between retinol intake and the risk of CKD in individuals with T2DM in humans. Multivariate logistic regression analysis demonstrated that individuals with higher retinol intake had a lower risk of CKD in individuals with T2DM.

Medical Nutrition Therapy (MNT) and management of risk factors, such as glucose, hypertension, hyperlipidemia, and uric acid, are currently the most widely used therapies for T2DM with CKD. Renin–angiotensin–aldosterone system (RASS) inhibitors, glucagon-like peptide-1 (GLP-1), mineralocorticoid receptor antagonists (MRA), and sodium-glucose transporter protein 2 (SGLT2) inhibitors are also administered and have proven renoprotective benefits^25^. Despite the availability of more treatment options, a significant number of individuals still progress to ESRD. More effective treatments are urgently being studied to prevent the progression of T2DM to suffer CKD. MNT is a cornerstone of multidisciplinary DM and CKD management. Dietary management of T2DM is known to be effective in slowing the incidence and progression of CKD, such as a low protein diet which is widely known to decrease proteinuria and slow the decline in e-GFR^26^.

Retinol, as the active form of vitamin A (VA), plays a crucial role in various biological functions, including proliferation, apoptosis, differentiation, and metabolism^9^. It must be supplied from the diet and is mainly found in the form of carotenoids and retinyl esters, which can be delivered to peripheral tissues via celiac remodeling^27^. The liver and adipose tissue are the main sites of retinol storage, accounting for 80–85% and 15–20% of total retinyl ester and retinol storage in the body, respectively^28^. Retinyl esters can be hydrolyzed to retinol and can be mobilized from the liver through retinol-binding protein 4 (RBP4), providing an additional source of retinol for additional peripheral tissues^10^. A study showed that individuals with T2DM over 40 years of age had similar vitamin A (VA) intake and plasma levels compared to controls South Asians with T2DM in the United States had lower total energy and retinol intake than controls^29^. A study showed that individuals with T2DM over 40 years of age had similar VA intake and plasma levels compared to controls^30^. However, individuals with diabetic retinopathy had a significantly higher dietary intake of retinol and retinol equivalents (*P <* 0.05) but lower serum retinol and RBP4 (*P <* 0.05) compared to the healthy population^17^. In this study, we found a significant difference in retinol intake between individuals with T2DM and chronic kidney disease (CKD) and those without CKD. Retinol intake is higher in older, male, Non-Hispanic White, and alcohol user in individuals with T2DM . This may be due to differences in eating habits between races and the fact that males and alcohol users tend to consume more food.

Moreover, an increasing number of studies suggest that retinol plays a crucial role in the incidence of diabetes and the progression of diabetic complications. For instance, dietary VA intake was associated with an increased risk of metabolic syndrome in Iranian women^31^.Higuchi K et al. showed a lower proportion of insulin resistance in subjects in the upper quartile of retinol compared to the lower quartile^15^. Similarly, Erikstrup C et al. found that lower plasma retinol concentrations in individuals with impaired glucose tolerance compared to normal in individuals with T2DM^16^. A prospective cohort study conducted in China with 17,111 participants demonstrated that a higher intake of retinol can help reduce the incidence of diabetes, particularly in males^32^. For studies on the risk of diabetic complications from retinol, Zhang C et al. showed that a higher dietary intake of retinol or retinol equivalents was associated with a lower risk of diabetic retinopathy, per 100 μg/day higher in dietary retinol equivalent intake was associated with 12% lower risk of diabetic retinopathy^17^. Retinol dehydrogenase-10 reduction-mediated disruption of retinol metabolism was found to promote diabetic myocardial ferroptosis in male mice, but this occurrence was ameliorated by retinol supplementation^18^.

In ob/ob type 2 diabetic mice, retinol metabolism-related genes were upregulated, suggesting that retinol metabolism may be involved in glomerular injury and the development of diabetic kidney disease^33^. The relationship between retinol and DKD has been increasingly studied in animal studies. Trasino SE et al.^21^ treated DKD mice with a Retinoic Acid Receptor β 2 Agonist and found that DKD mice in the experimental group showed milder pathological changes (tubular lipid droplets, loss of podocytes (POD), endothelial cell collapse, thylakoid expansion, and glomerular basement membrane thickening) and lower expression of myofibroblast markers-smooth muscle actin (-SMA) and type IV collagen (-Col IV) compared to controls. Diabetes-induced increases in cytokines such as interleukins, tumor necrosis factor-alpha (TNF-α), transforming growth factor-beta 1 (TGF-β1), chemokines (CCL2, CCL20, CXCL5, and CXCL7), adhesion molecules (ICAM-1 and L-selectin) and growth factors (GM-CSF, VEGF, PDGF) in the glomerulus and proximal tubules, and these changes were significantly improved by all-trans retinoic acid treatment^34^. All of the aforementioned studies suggest that retinol may play a crucial role in the risk of CKD. However, the relationship between retinol intake and the risk of CKD in individuals with T2DM remains unclear. Our study aims to fill this gap by demonstrating that higher retinol intake is associated with a lower risk of CKD in T2DM individuals. This finding provides a theoretical basis for exploring the use of retinol to prevent CKD in this population.

The mechanism behind why individuals with T2DM who consume more retinol have a lower risk of CKD requires further investigation. Retinol has been reported to possess antioxidant properties that scavenge free radicals, inhibit peroxidation, and maintain homeostasis between oxidants and antioxidants^35^. Additionally, retinol has anti-inflammatory abilities, as it impedes the expression of various pro-inflammatory cytokines such as TNF-α, IL-6, and IL-12, etc.^36^ Intrarenal oxidative stress plays a critical role in the progression of DKD, and antioxidant therapy has been shown to be beneficial in slowing its progression^37^. Similarly, inflammation plays an important role in the development and progression of T2DM with CKD, and anti-inflammatory therapy is a promising approach to exploring its treatment^38^. The aforementioned antioxidant and anti-inflammatory activities may be involved in retinol's nephroprotective efficacy. In animal studies, RA has been shown to inhibit the expression of pro-inflammatory cytokines such as tumour necrosis factor-α, tumour growth factor β and interleukin 6, as well as the downregulation of Toll-like receptor 4 signallings in diabetic rats^34^. It has also been demonstrated that diabetic rats treated with RA have improved levels of oxidative stress^41^. However, in our study, linear regression models showed that retinol had no significant effect on ACR, e-GFR, serum albumin, hemoglobin, or HbA1c. Therefore, further basic experimental studies and prospective observational studies are needed to investigate.

However, it is important to note that this study has some limitations. Firstly, due to its cross-sectional design, it is not possible to establish a cause-and-effect relationship between retinol intake and the risk of CKD in T2DM. Additionally, relying on two 24-h dietary recall interviews may not accurately represent long-term dietary intake. Lastly, there were some factors that were not controlled for, which could have influenced the results.

In conclusion, our study suggests that lower retinol intake is an independent risk factor for the risk of CKD in individuals with T2DM. This finding highlights the importance of moderate retinol intake in T2DM individuals and provides a basis for further exploration of retinol as a potential preventative treatment for CKD in this population.

## Supplementary Information

Supplementary Information 1.

Supplementary Figure 1.

## Data Availability

Some or all datasets generated during and/or analyzed during the current study are not publicly available but are available from the corresponding author on reasonable request.
