# Supplementary material for: Retinol intake is associated with the risk of chronic kidney disease in individuals with type 2 diabetes mellitus: results from NHANES
Source: Sci Rep. 2023 Jul 18;13:11567. doi: 10.1038/s41598-023-38582-z (PMC10354112; doi:10.1038/s41598-023-38582-z)
Supplement: Supplementary file 1 — Supplementary Information 1. [file 41598_2023_38582_MOESM1_ESM.docx]

**Supplementary -table 1. Definitions/criteria of some diagnoses**

| Variables | Definitions/criteria |
| --- | --- |
| Smoker | Smoking more than 100 cigarettes in previous and now. |
| Alcohol user ^1^ | ≥2 drinks per day for females, ≥3 drinks per day for males, or binge drinking ≥2 days per month.  Binge drinking (≥4 drinks on the same occasion for females, ≥5 drinks on the same occasion for males) on 5 or more days per month. |
| Hypertension ^2^ | 1. Self-reported hypertension diagnosis, (2) Use of anti-hypertensive medication, (3) Average systolic blood pressure (SBP) > 140 mmHg, (4) Average diastolic blood pressure (DBP) > 90 mmHg, meet any of the above conditions. |
| Anemia ^3^ | ≥120g/L for women (15 years of age and above), ≥130g/L for men (15 years of age and above). |
| Hyperlipidemia | (1) Triglyceridemia ≥ 150 mg/dl; (2) Hypercholesterolemia: a) total cholesterol ≥ 200 mg/dl, b) low-density lipoprotein ≥ 130 mg/dl), c). high-density lipoprotein (< 40 mg/dl, male; < 50 mg/dl, female), meet any of the above conditions; (3) Use of lipid-lowering drugs; meet any of the above conditions. |

**Supplementary -table 2. Cox-regression analysis of risk factors for the development of CKD in T2DM**

| Variables | Unadjusted | | | Model 3 | | |
| --- | --- | --- | --- | --- | --- | --- |
|  | OR | (95%CI) | P-value | OR | (95%CI) | P-value |
| Retinol |  |  |  |  |  |  |
| Lower retinol intake | ref | ref |  | ref | ref |  |
| Higher retinol intake | 0.79 | (0.63,0.99) | 0.04 | 0.74 | (0.56,0.98) | 0.04 |
| Age | 1.05 | (1.04,1.06) | <0.001 | 1.05 | (1.04,1.07) | <0.001 |
| Sex |  |  |  |  |  | 0.20 |
| Female | ref | ref |  | ref | ref |  |
| Male | 1.09 | (0.85,1.39) | 0.51 | 1.38 | (1.05,1.82) | 0.02 |
| BMI |  |  |  |  |  |  |
| Under weight | ref | ref |  | ref | ref |  |
| Normal weight | 0.41 | (0.12,1.43) | 0.16 | 0.31 | (0.08,1.20) | 0.09 |
| Obesity | 0.35 | (0.11,1.19) | 0.09 | 0.24 | (0.06,0.93) | 0.04 |
| Over weight | 0.41 | (0.12,1.38) | 0.15 | 0.31 | (0.09,1.28) | 0.11 |
| Race |  |  |  |  |  |  |
| Mexican American | ref | ref |  | ref | ref |  |
| Non-Hispanic Black | 1.16 | (0.90,1.51) | 0.25 | 0.72 | (0.49,1.05) | 0.72 |
| Non-Hispanic White | 1.20 | (0.93,1.54) | 0.15 | 0.89 | (0.61,1.29) | 0.89 |
| Other Hispanic | 0.70 | (0.50,1.00) | 0.05 | 0.58 | (0.36,0.94) | 0.58 |
| Other Race - Including Multi-Racial | 1.07 | (0.75,1.53) | 0.70 | 0.91 | (0.54,1.53) | 0.91 |
| Hypertension [n (%)] |  |  |  |  |  | 0.03 |
| No | ref | ref |  | ref | ref |  |
| Yes | 2.27 | (1.80,2.87) | <0.001 | 1.37 | (1.04,1.81) |  |
| Alcohol user |  |  |  |  |  | 0.09 |
| No | ref | ref |  | ref | ref |  |
| Yes | 0.70 | (0.55,0.89) | 0.004 | 0.77 | (0.57,1.04) |  |
| Smoke |  |  |  |  |  | 0.03 |
| No | ref | ref |  | ref | ref |  |
| Yes | 1.34 | (1.06,1.68) | 0.01 | 1.37 | (1.04,1.81) |  |
| **HbA1c** | 1.19 | (1.11,1.27) | <0.001 | 1.30 | (1.19,1.42) | <0.001 |
| Serum albumin | 0.90 | (0.87,0.93) | <0.001 | 0.91 | (0.79,0.95) | 0.003 |
| Energy intake | 1.00 | (1.00,1.00) | <0.001 | 1.00 | (1.00,1.00) | 0.19 |

OR, odds ratio; CI, Confidence interval; BMI, Body Mass Index; CKD, chronic kidney disease; T2DM, type 2 diabetes mellitus**.**

**Supplementary -table 3. Associations between retinol intake and the development of CKD in patients with T2DM**

| Variables | Unadjusted | | Model 1**^a^** | | Model 2**^b^** | | Model 3**^c^** | |
| --- | --- | --- | --- | --- | --- | --- | --- | --- |
|  | 95% CI | P-value | 95% CI | P-value | 95% CI | P-value | 95% CI | P-value |
| Lower retinol intake | ref |  | ref |  | ref |  | ref |  |
| Higher retinol intake | 0.79(0.63,0.99) | 0.04 | 0.71(0.55,0.92) | 0.01 | 0.73(0.56,0.95) | 0.02 | 0.74(0.56,0.98) | 0.03 |
| Per-SD increment of retinol intake | 0.88(0.79,0.98) | 0.02 | 0.83(0.74,0.94) | 0.004 | 0.82(0.72,0.93) | 0.002 | 0.84(0.72,0.97) | 0.02 |

**Model 1^a^** adjusted for baseline age, sex, race, BMI; **Model 2^b^** adjusted for covariates in model 1 plus smoke (‘yes’ or ‘no’), alcohol use (‘yes’ or ‘no’), hypertension (‘yes’ or ‘no’). **Model 3^c^** adjusted for covariates in model 2 plus anemia (‘yes’ or ‘no’), anti-diabetic drugs, HbA1c, serum albumin. OR, odds ratio; CI, Confidence interval; BMI, Body Mass Index; CKD, chronic kidney disease; T2DM, type 2 diabetes mellitus**.**

**
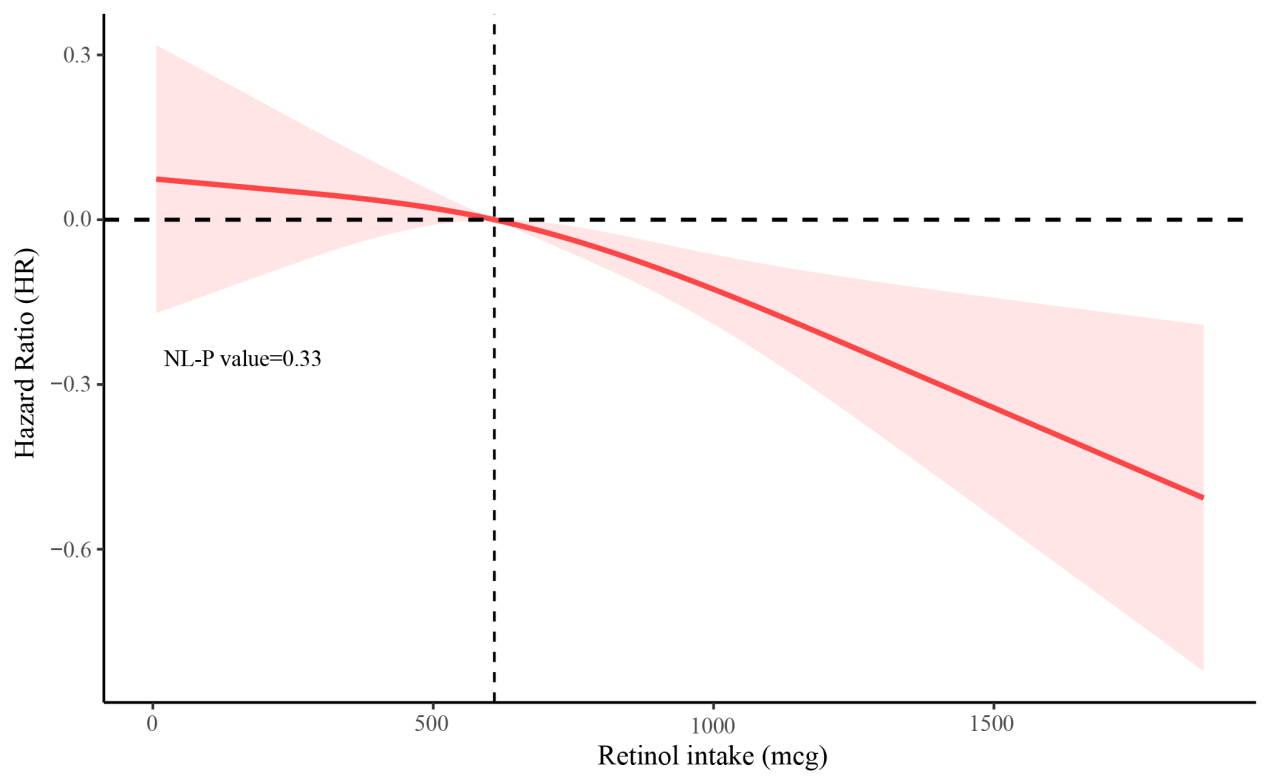
**

**Supplementary Figure 1.** Association between retinol intake and the occurrence of kidney injury in individuals with DKD based on restricted cubic spline plot.

**References**

1. Rattan P, Penrice DD, Ahn JC, et al. Inverse Association of Telomere Length With Liver Disease and Mortality in the US Population. *Hepatology communications* 2022; **6**(2): 399-410.

2. Whelton PK, Carey RM, Aronow WS, et al. 2017 ACC/AHA/AAPA/ABC/ACPM/AGS/APhA/ASH/ASPC/NMA/PCNA Guideline for the Prevention, Detection, Evaluation, and Management of High Blood Pressure in Adults: A Report of the American College of Cardiology/American Heart Association Task Force on Clinical Practice Guidelines. *Journal of the American College of Cardiology* 2018; **71**(19): e127-e248.

3. WHO. Haemoglobin concentrations for the diagnosis of anaemia and assessment of severity. Vitamin and Mineral Nutrition Information System. Geneva, World Health Organization, 2011 (WHO/NMH/NHD/MNM/11.1).
