# Supplementary figures and images for: Retinol intake is associated with the risk of chronic kidney disease in individuals with type 2 diabetes mellitus: results from NHANES
Source: Sci Rep. 2023 Jul 18;13:11567. doi: 10.1038/s41598-023-38582-z (PMC10354112; doi:10.1038/s41598-023-38582-z)

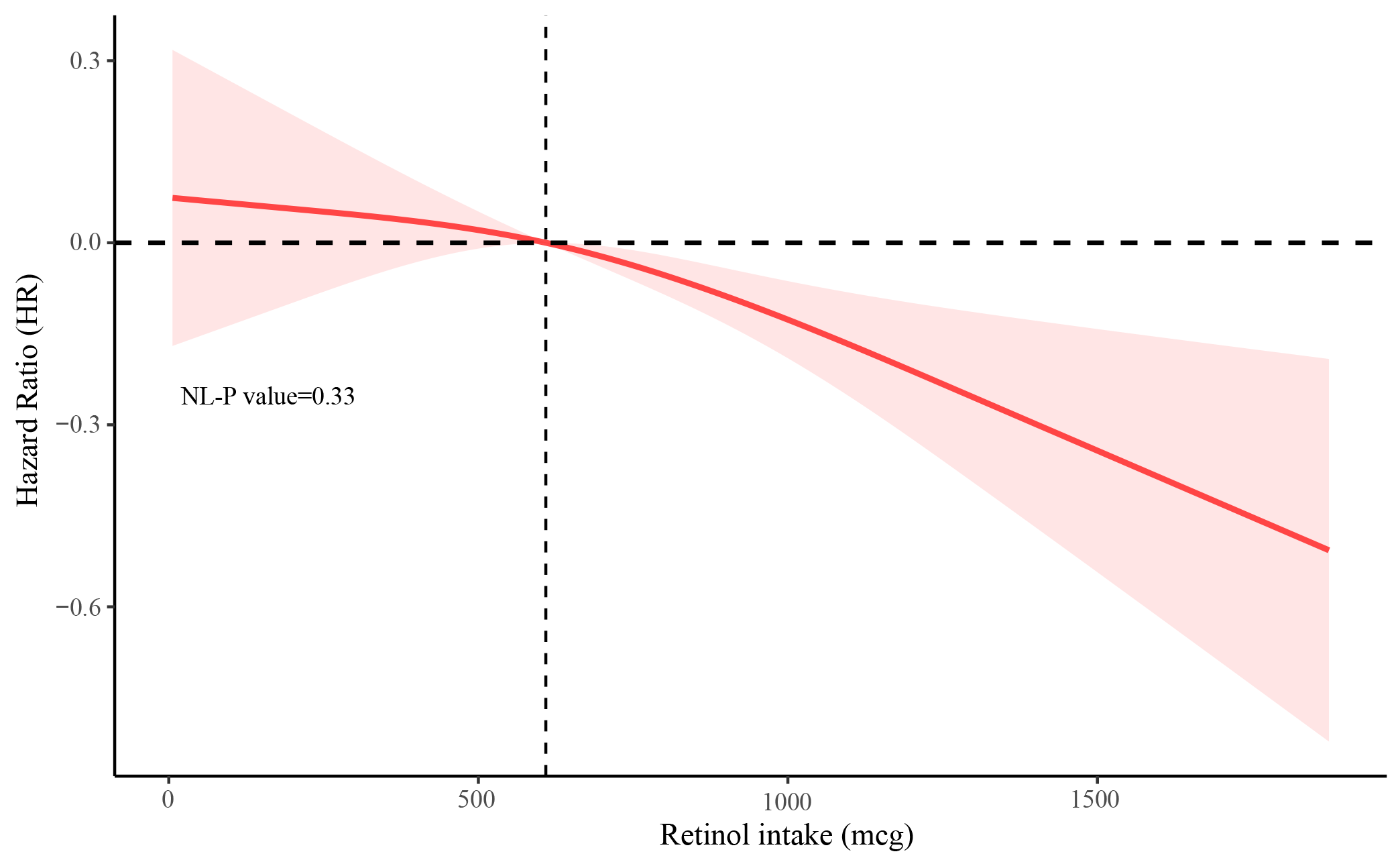

Supplement: Supplementary file 2 — Supplementary Figure 1. [file 41598_2023_38582_MOESM2_ESM.tif]
